# Energy and Macronutrient Dietary Intakes of Serbian Adults 18–64 Years Old: EFSA EU Menu Food Consumption Survey in Serbia (2017–2022)

**DOI:** 10.3390/foods14071228

**Published:** 2025-03-31

**Authors:** Jelena Milešević, Milica Zeković, Ivana Šarac, Marija Knez, Irena Krga, Marija Takić, Jasmina Debeljak Martačić, Vuk Stevanović, Nevena Vidović, Slavica Ranković, Agnes Kadvan, Mirjana Gurinović

**Affiliations:** 1 Center of Research Excellence in Nutrition and Metabolism, Group for Nutrition and Metabolism, Institute for Medical Research, National Institute of Republic of Serbia, University of Belgrade, 11000 Belgrade, Serbia; milica.zekovic@imi.bg.ac.rs (M.Z.); ivana.sarac@imi.bg.ac.rs (I.Š.); marija.knez@imi.bg.ac.rs (M.K.); irena.krga@imi.bg.ac.rs (I.K.); marija.takic@imi.bg.ac.rs (M.T.); jasmina.martacic@imi.bg.ac.rs (J.D.M.); vuk.stevanovic@imi.bg.ac.rs (V.S.); nevena.vidovic@imi.bg.ac.rs (N.V.); slavica.rankovic@imi.bg.ac.rs (S.R.); mirjana.gurinovic@gmail.com (M.G.); 2 Capacity Development in Nutrition CAPNUTRA, 11000 Belgrade, Serbia; k_agi@yahoo.com

**Keywords:** food consumption, dietary assessment, nutritional status, dietary intake, dietary survey

## Abstract

This study offers a comprehensive analysis of the anthropometric and nutritional status among Serbian adults aged 18–64 years, using the data from the EFSA EU Menu food consumption survey conducted between 2017 and 2022. Based on a nationally representative sample of 1139 participants, this research utilized validated 24 h dietary recalls and anthropometric measurements. The results indicate significant dietary imbalances, characterized by a heavy reliance on energy-dense foods, such as grains, fats, and meat, with an underrepresentation of fruits, vegetables, and dairy. Men exhibited a higher total energy intake, obtaining more energy from meat and fats, while women consumed more fruits and vegetables but often did not meet the recommended protein intake. Approximately 67.8% participants had a insufficient dietary fiber intake, and 15.4% did not meet the protein recommendations, particularly women. Anthropometric measurements showed a high prevalence of overweight and obesity, especially among men. These findings highlight critical deficits in dietary adequacy and, from a clinical practice perspective, underscore the necessity for the formulation of policies, targeted public health strategies aimed at improving dietary habits, and provide evidence for the development of national dietary guidelines and clinical guidelines to support preventive healthcare strategies, especially in the management of diet-related conditions, such as obesity and metabolic disorders.

## 1. Introduction

Nutritionally adequate, diverse, and balanced diets are important for health promotion and maintenance throughout one’s life course. Nevertheless, current dietary patterns are commonly unhealthy, unsustainable, and inequitable for many populations, and present a substantial proportion of the global burden of disease and death [1]. The analysis of the impact of the inadequate consumption of major foods and nutrients across 195 countries on non-communicable disease (NCD) mortality and morbidity identified diets high in salt, fat, and sugar, and low in whole grains, fruits, vegetables, nuts and seeds, and omega-3 fatty acids as the leading risk factors for mortality, each contributing to more than 2% of global deaths. Non-optimal intake of three key dietary factors (whole grains, fruits, and sodium) accounted for more than 50% of deaths and 66% of disability-adjusted life years (DALYs) attributable to diet [2,3,4,5]. As the COVID-19 crisis has shown, untreated nutrition-related comorbidities, such as arterial hypertension, chronic obstructive bronchitis, immuno-suppression, CVDs, diabetes mellitus type 2, and obesity, exacerbated treatment of the infection and were the highest risk factors for fatal outcomes. Moreover, previous studies demonstrated that there is positive correlation with adherence to healthy diet (such as the Mediterranean diet) and lifestyle (including physical activity, social activities, mental health, etc.) and metabolic clinical parameters, such as Creatinine, Fasting Glucose (FG), Glycosylated Hemoglobin (HbA1c), High-Density Lipoprotein (HDL), Low-Density Lipoprotein (LDL), Total Cholesterol (TC), Diastolic (Arterial) Blood Pressure (DBP), and Systolic (Arterial) Blood Pressure (SBP) levels, and anthropometric ones—body mass index (BMI) [6].

Nutritional intake and related dietary behaviors are influenced by a variety of factors, including socioeconomic status, cultural practices, environmental conditions, and personal preferences. These interactively influence both individual and population dietary patterns. In Serbia, the relationship between nutrition and non-communicable diseases is significantly influenced by socioeconomic inequalities [7,8].

The availability of the standardized and harmonized individual food consumption data from national dietary surveys are essential for the dietary quality assessment, longitudinal monitoring, and dietary shift estimation for evidence-based policy making, to identify the priorities of public health and to support evidence-based interventions [9,10,11]. They provide detailed and representative data on the intake of foods and nutrient adequacy, as well as their related health risks [12].

Historically, the data on adult dietary intake and nutritional status in Serbia are limited and inconsistent, pointing to a strong need for robust harmonized research methodologies [13]. A Yugoslav study of atherosclerosis precursors in schoolchildren in Serbia was the first and until now the only national dietary survey in Serbia that conducted comprehensive food consumption collection using validated dietary assessment methods (incl. energy and nutrient intake of families and children) [14,15]. In this study, conducted on children and their families in 1998 and 2003, a seven-day consumer nutrition survey was conducted where the data on the daily amount of food consumed in grams during seven consecutive days in a household was collected. Furthermore, the Family Nutrition Survey software (v.1.) was developed and used to determine the average energy and nutrient intake in school-aged children of 10 (*n* = 1319) and 15 years of age (*n* = 966 children; 458 girls and 502 boys) in 10 and 13 YUSAD regional centers for the study in Serbia. The study included a collection of the anthropometric measurements of children: height, weight, waist circumference, subscapular skinfold, and blood pressure [14,15]. This methodology could not address the precise estimates of dietary intake of specific population groups as the data on individual food consumption was not collected, yet family-based cumulative food consumption per day. Learning from this approach, further development of the dietary intake assessment methodology and research infrastructure (capacity development in nutritional research, food composition database, software, etc.) took place. After this study, no national representative study that assessed the nutritional status of any and particularly the adult population in Serbia was conducted until 2017. A similar situation was identified in other countries in the region (Bosnia and Herzegovina, Montenegro, and North Macedonia) [16].

To address this situation, we have been working on building and improving the nutritional research capacity for a decade, developing the food composition and recipe database [16] and nutritional assessment tools [13]. The comprehensive dietary assessment tool, Diet Assess and Plan (DAP), was tested and validated at the national and regional levels [17] harmonized with internationally recognized methodological standards and evaluated in the European Food Safety Authority (EFSA) project “Dietary Monitoring Tools for Risk Assessment” [18]. EFSA has directed the harmonization of food consumption data collection under the EU Menu framework in Europe, by providing a standardized protocol that allows for cross-country comparisons and supports the generation of high-quality, comparable, harmonized food consumption data. As a result of these endeavors and prospects, we gained the opportunity to take on the National Food Consumption Survey that was carried out within the EFSA program, in which we adapted our research infrastructure following the established EU Menu methodology, guidelines, and protocols [12]. This survey was an important step toward producing valid food consumption data not only in Serbia, but in other Balkan countries (led by national appointed institutions) that used the same program scheme and methodology, and finally filling the gap in the harmonization of food consumption data in this part of Europe. This study was conducted simultaneously in four Balkan countries with national adaptations (distribution of the recruiting sample across regions/cantons in a particular country) that allowed capturing seasonal, cultural, religious, and geographically influenced nutritional differences [9,10]. Moreover, in the data collection process, the Balkan regional food composition database was enlarged with the collection of national, frequently consumed traditional foods and recipes to allow precise nutritional assessments. Moreover, a dietary survey on children aged 1–9 years old was also conducted following a slightly different, age-adapted EU Menu methodology, of which the analysis has been previously published [19,20].

### Aims

The primary objective of this study was to assess the nutritional and anthropometric status of Serbian adults aged 18–64 years, utilizing the data collected through the EFSA EU Menu food consumption survey conducted from 2017 to 2022.

The secondary objectives were as follows:

- To provide detailed evidence on the energy and macronutrient intake patterns among Serbian adults, identifying the key nutritional imbalances and gender-based disparities in dietary habits.
- To evaluate the adequacy of protein, fat, carbohydrate, and fiber intake in relation to the international dietary recommendations, including those of EFSA and the US Dietary Guidelines.
- To assess the prevalence of overweight, obesity, and inadequate nutrition, particularly the underconsumption of essential micronutrients, such as fruits, vegetables, and fiber.
- To contribute to the ongoing harmonization of dietary data across European countries, enhancing the comparability of Serbia’s dietary data with other European nations, and supporting the development of targeted public health policies.
- To provide actionable insights for the development of national dietary guidelines and to inform public health strategies aimed at improving the dietary habits of Serbian adults, with particular attention being paid to gender-specific needs and nutritional interventions.

## 2. Materials and Methods

The methodology of the work is presented following the STROBE (Strengthening the Reporting of Observational Studies in Epidemiology) guidelines, to ensure transparency and consistency in the reporting of this cross-sectional study. The key elements of the methodology are study design, setting, participants, variables, data collection, and statistical analysis [21].

### 2.1. Study Design

The Serbian Adult Food Consumption Survey was conducted as part of the broader national dietary survey, assessing the nutrient intakes and related health indicators among the adult population from 10 to 74 years of age. Conducted in alignment with the standardized protocols established by the EFSA under the EU Menu methodology [12], this study ensured methodological robustness, cross-country comparability, and data integrity. The data collection was in the range of 2017–2022, encompassing a nationally representative cohort of individuals aged 18–64 years.

### 2.2. Setting

The survey was conducted across Serbia, utilizing the national population registry as the primary sampling frame. Participants were recruited from private households, with sampling stratified by age, gender, and residential region (Belgrade, AP Vojvodina, Southeastern Serbia, and Šumadija and West Serbia) to ensure a representative sample. The data were collected during all four seasons to account for seasonal variations in diet, and the data were evenly distributed across weekdays, national holidays, and festive periods to reflect diverse consumption behaviors. Data collection occurred between 2017 and 2022, with fieldwork conducted in compliance with the ethical standards set by the Institutional Ethics Committee (Approval number: EO123/2017, issued on 8 December 2017).

### 2.3. Participants

The target population included adults aged 18–64 years residing in Serbia. Participants were systematically selected from private households based on stratification criteria to ensure representativeness of the population. The final sample consisted of 1139 adults (576 women, 563 men). The eligibility criteria required participants to be Serbian nationals or residents and able to provide informed consent. All individuals who met these criteria were invited to participate, with written informed consent obtained prior to inclusion in this study. Institutionalized individuals were excluded from participation as their nutrition was organized within the institution where they were cared for, and did not reflect individual dietary preferences.

### 2.4. Variables

Outcomes: The primary outcomes of interest were dietary intake (energy, macronutrients, and micronutrients) and anthropometric measures (body weight, height, waist circumference, and BMI).

Exposures: Key exposures included dietary patterns, physical activity levels, and socio-demographic variables, such as age, gender, and educational level.

Predictors: This study examined predictors such as regional and seasonal variations in diet, as well as the intake of specific food groups (e.g., fruits, vegetables, grains, and dairy).

Confounders: Potential confounders included socioeconomic status, physical activity levels, and chronic health conditions (e.g., obesity and hypertension).

Effect modifiers: Gender and age were considered potential effect modifiers in the relationship between dietary intake and health outcomes.

### 2.5. Data Sources/Measurement

*Dietary Intake*: Dietary data were collected using two non-consecutive 24 h dietary recalls spaced at least one week apart. The multiple-pass interview method was used to ensure completeness and accuracy. Data on food items, meal types, preparation methods, and portion sizes were recorded. A photographic Food Atlas tailored to the Balkan region was used to support portion size estimations [22]. Nutrient intake was calculated using the Serbian Food Composition Database (FCDB) [23], and foods were categorized using the FoodEx2 system to enable cross-national comparability.

*Physical Activity:* Data on physical activity were collected using the International Physical Activity Questionnaire—Short Form (IPAQ-SF), categorizing participants into low, medium, and high physical activity levels [24,25].

*Anthropometric Measures:* Weight, height, and waist circumference were measured using calibrated equipment. BMI was calculated using standard formulas, and participants were categorized based on the WHO BMI classifications [26].

### 2.6. Bias

To address potential sources of bias, stratified sampling was employed to ensure representativeness across gender, age, and region. Seasonal fluctuations in dietary patterns were accounted for by spreading the data collection across all seasons. Fieldworkers underwent extensive training, and data collection tools were pilot tested to minimize errors. Regular audits were conducted to ensure adherence to standardized protocols.

### 2.7. Study Size

The sample size was determined based on demographic projections and the need for a nationally representative sample. A total of 1139 participants were included, with approximately equal gender distribution and sufficient regional representation to ensure the generalizability of the findings.

### 2.8. Quantitative Variables

Quantitative variables, including energy intake (kcal), macronutrient intake (grams), and anthropometric measurements (weight, height, and waist circumference), were analyzed using the median and interquartile range (IQR) due to the non-normal distribution of the data. Subgroup analyses were performed by gender and age group, with appropriate adjustments for confounders.

### 2.9. Statistical Methods

*Confounding Control:* Statistical analysis was performed using SPSS software (v. 26.0, USA). The Mann–Whitney U test was used for non-normally distributed data, while Pearson’s chi-squared or Fisher’s exact tests were used for categorical variables. Statistical significance was set at *p* < 0.05.

*Subgroup Analysis and Interactions:* Gender and age-specific subgroup analyses were conducted to assess potential differences in dietary intake and health outcomes. Interaction terms were included to examine the effects of gender and age on dietary patterns and nutritional status.

*Missing Data:* Missing data were handled using multiple imputation methods to minimize bias and ensure the robustness of the findings. *Cross-sectional Analysis:* Data analysis took into account the stratified sampling design by using appropriate weightings for each subgroup. The analysis adjusted for the complex survey design to account for potential clustering effects and ensure accurate estimates.

*Sensitivity Analyses:* Sensitivity analyses were conducted to examine the robustness of the results, including variations in seasonal data collection and the impact of different dietary recall methods.

## 3. Results

A total of 1139 eligible participants (576 women, 563 men) from Serbia took part in the EFSA EU Menu-based food consumption survey (2017–2022). The overall response rate was ~80%; 1155 adults aged 18 to 64 were recruited and the response rate was 82.74% among female and 79.39% among the male population, with no significant gender or age difference (data published elsewhere) [20]. The median age was ~41–43 years, and participants were distributed among age groups in line with the demographic situation according to the state Census from 2011: 18–24 (~13%), 25–44 (~41%), and 45–64 (~46%) years. Regional representation was balanced, with the Šumadija and Western Serbia region having the largest subgroup (~28%). Most participants were urban residents (~86%), mirroring Serbia’s urban–rural demographics.

Most of the sample has a Serbian nationality (~93%) and Orthodox religion, with similar religious affiliations among genders. Marital status differed significantly: more men were single (~36% vs. ~30%) and fewer divorced (~4% vs. ~6%). Both genders had a median household size of three members (IQR: 2–4). Men were more represented in vocational and post-secondary education, while women had higher qualifications at Master’s or Doctoral levels (~16% vs. ~10%).

Employment rates were higher for men (~76% vs. ~66%), while women more often engaged in unpaid work. Women had a higher prevalence of chronic illnesses (30.6% vs. 18.5%) and reported endocrine and genitourinary disorders more frequently. Men reported higher physical activity levels (~13% vs. ~10%). Most dietary patterns were conventional (94%), but health-related adjustments were more common among women (~8% vs. ~3%). Seasonal participation showed no significant gender differences (Table 1). This comprehensive demographic profiling provides a valuable context for understanding dietary and health outcomes among Serbian adults.

### 3.1. Anthropometric Data and Nutritional Status

This study revealed significant gender differences in anthropometric measurements and nutritional status. The median height for males and females was, respectively, 174 cm and 168 cm, while body mass was 85 kg and 65 kg. Males exhibited a significantly greater median BMI, 25.9 kg/m^2^ (in the range of overweight), while in females it was 23.1 kg/m^2^ (in the range of normal weight) (*p* < 0.001). Nutritional status, classified by BMI [26], showed that ~50% of participants had a normal weight in the overall sample, but there were huge gender differences: only ~37% of men but ~64% of women had a normal weight. More men were overweight (~47%) or obese (~15%) compared to women (~24% and ~9%, respectively, *p* < 0.001). Underweight prevalence was higher in women (~4%) than in men (~1%, *p* < 0.001) (Table 2).

### 3.2. Daily Energy and Macronutrient Intake Across Gender

Table 3 shows significant gender differences in energy and macronutrient intake. Median energy intake was higher in men (2800 kcal/day) than women (2070 kcal/day). Men also consumed more carbohydrates (~250 vs. ~200 g), protein (~105 vs. ~75 g), fat (~140 vs. ~100 g), and protein per kg of body mass (1.2 vs. 1.1 g) (*p* < 0.001 for all). The median daily protein intake exceeded the EFSA Population Reference Intakes (PRI) of 0.83 g/kg body weight for most participants, though ~17% did not meet this threshold, with more women (~21%) than men (~14%, *p* = 0.011) being the case. [Note: for the USA Recommended Dietary Allowances (RDA) of 0.8 g/kg body weight, overall ~16% did not meet the threshold, ~18% of women and ~13% of men, *p* = 0.011)]. In women, median carbohydrate and fiber intake were for ~30% and ~20% lower, respectively, while fat intake was ~50% higher than the recommended amount, while protein intake and total energy intake were aligned with the recommendations. For men, total energy intake was ~150–200 kcal above the recommendations, and fat intake was ~60% higher, while carbohydrate and fiber intake were ~10% and ~40% lower than recommended.

In women, protein intake per kg of body weight was not related to the level of education, nor was total protein intake. Among men, those with a lower protein intake per kg of body weight (0.8 g/kg body weight) had a lower level of education (Mann–Whitney, *p* = 0.029), and the share of protein in the structure of the daily meal was significantly positively correlated with the level of education (Spearman rs = 0.121, *p* = 0.004), while the share of carbohydrates was inversely correlated (rs = −0.121, *p* = 0.004).

Macronutrient distribution as a percentage of total energy intake (%TE) revealed distinct patterns: carbohydrate intake was higher in women (~38% TE vs. ~36% TE in men, *p* < 0.001), while men had greater contributions from fat (~45% TE vs. ~44% TE, *p* = 0.011). Protein intake constituted ~15% TE overall, slightly higher in men (~15% TE vs. ~14% TE in women, *p* = 0.001). Notably, fiber intake fell below the EFSA recommended threshold of 25 g/day for 68% of the population, with women having a higher prevalence of an inadequate intake (~71% vs. ~65%, *p* = 0.026). [Note: the US recommendations advise a much higher fiber intake: 29 g for women and 37 g for men].

### 3.3. Energy Intake Categories and Macronutrient Distribution Compared to the Dietary Guidelines

Since there are no national dietary guidelines in Serbia, we compared the findings with the EFSA DRV [27] and US DRI [28] guidelines for energy intake and macronutrient distribution. The data again reveal significant deviations among Serbian adults comparing to the dietary guidelines (Table 4).

The total energy intake distribution shows that ~18% of participants fall below the recommended thresholds (<1600 kcal/day for women and <2000 kcal/day for men), with women more likely to fall into this category (~20% vs. ~15%, *p* < 0.001). Conversely, men were more likely to exceed the highest energy intake thresholds (≥3000 kcal/day), with ~42% of men compared to ~27% of women in this category.

Carbohydrate intake was disproportionately low in the overall population, with 86% of participants consuming less than 45% of total energy from carbohydrates, significantly diverging from both the EFSA and US dietary recommendations (45–60%, i.e., 45–65% of total energy, respectively). Women were slightly more likely to meet the carbohydrate recommendations (~18% vs. ~10% of men, *p* < 0.001). Protein intake was largely within the recommended range (10–35% of total energy), but a small fraction of participants consumed less than 10%, more commonly women (~4% vs. ~1% of men, *p* = 0.001).

Fat intake exceeded the EFSA and US dietary guidelines (>35% of total energy) for almost all (90%) of the participants, with no significant gender difference observed. Nevertheless, when the newer WHO&FAO recommendations from 2024 were applied (15–30% TE) [29,30], 97% of the population exceeded the limit in both genders.

### 3.4. Daily Food Group Consumption

The dietary habits of Serbian adults, as detailed in Table 5, indicate significant discrepancies from the Dietary Guidelines for Americans (2020–2025) [31], underscoring the necessity for enhanced nutritional balance. The median daily intake of vegetables was 274 g, which nearly meets the recommended range of 300–400 g/day; however, there is often a lack of variety (the main sources were potato, tomato, and beans). Conversely, fruit consumption was notably low, with a median intake of 124 g/day, significantly below the recommended 200–300 g/day. Although women consumed more fruits and vegetables than men, neither gender met the recommended intake. The most consumed fruit were apples.

A median intake of dairy was ~190 g in females and ~230 g in males, mostly from semi-skimmed yogurt and white full-fat cheese, while grains intake was ~170 g/day in females and ~240 g/day in males, but almost completely from refined grains.

A median intake of meat and meat products was high, especially in males, ~215 g, while in females it was ~125 g, both exceeding the EAT-Lancet Commission recommendations for a healthy diet (14–29 g/day) [32] and the Global Burden of Disease target reference for a healthy diet (18–27 g/day) [2]. In contrast, fish and seafood, essential for omega-3 fatty acids, were virtually absent from the diet. Additionally, sugar, sweets, added oils, and fat intakes were notably high. Except for fish/seafood, nuts/seeds, and sweets, the amount of all other food groups eaten was higher in men, as expected.

### 3.5. Energy and Proximates Intake by Food Groups

The contribution of various food groups to total energy intake among Serbian adults complement the insights from Table 5. 

**Figure 1:**
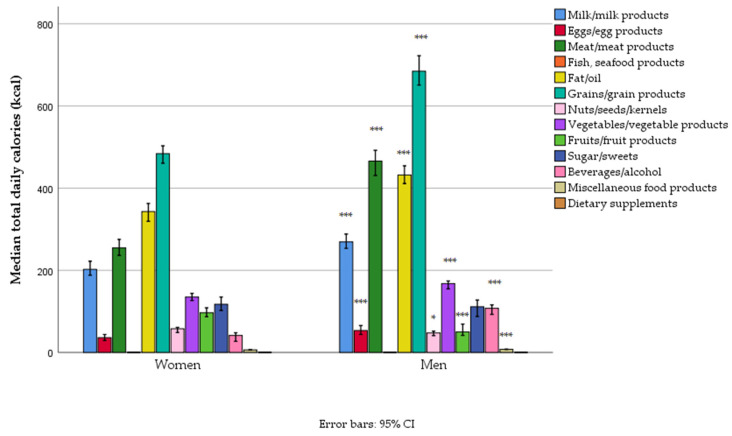
Energy intake from different food groups in women and men in a nationally representative sample of 18–64-year-old adults (*n* = 1139) living in Serbia (* *p* < 0.05, *** *p* < 0.001).

The energy intake among Serbian adults, organized by macronutrient sources and food groups, reveals critical dietary imbalances. Energy contributions were predominantly by grains, added oils and fats, meat, and dairy, while fish, fruits, vegetables, and nuts/seeds contributed less (Table 6, Figure 1).

The main energy sources were refined wheat flour products and sunflower oil. Polished rice was the second most-consumed cereal. From added fat and oils, olive oil, pork lard, and margarines also contributed; from meat, mostly pork meat, pork bacon, less beef, and chicken contributed; from dairy, it was mostly semi-skimmed yogurt, white soft full-fat cheese, semi-skimmed milk, and ripe, hard, white full-fat cheese; from vegetables, it was mostly potato and less legumes (beans); from fruit, apples and bananas; from sweets, sugar, chocolate, and biscuits; and beer and soda drinks also significantly contributed. 

The energy contribution of grains, milk, and eggs was not different between women and men. For men, meat and beverages/alcohol contributed more, while in women, other food groups had a higher energy contribution (Table 6, Figure 1). However, in absolute terms (kcal/day), the intake was higher in men for all food groups, except for fish and sweets (Figure 1, Supplementary Table S1).

**Figure 2:**
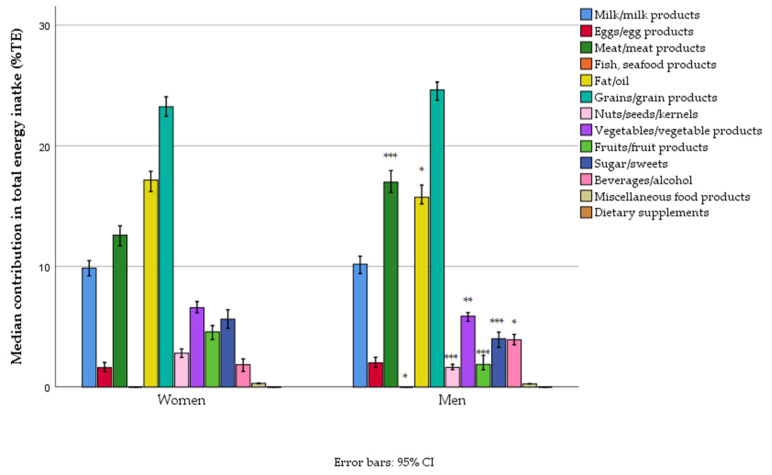
Different food groups’ contribution to daily energy intake in women and men in a nationally representative sample of 18–64-year-old adults (*n* = 1139) living in Serbia (* *p* < 0.05, ** *p* < 0.01, and *** *p* < 0.001).

The findings highlight a disproportionate reliance on grains, meat, and fats, which contribute significantly to total energy, while fruits and vegetables remain underrepresented. Grains and grain products accounted for the largest share of energy intake (~24% TE), consistent across genders, although men exhibited slightly higher consumption levels. Meat and meat products were the second-largest contributor (median: 14.5% TE), significantly higher in men than women (Table 6). Fats and oils contributed ~16% of total energy (TE), with women having a slightly higher intake. Fruits and vegetables had low contributions (~3% and ~6% TE, respectively), where women had a marginally higher intake of fruits and vegetables compared to men. Milk and dairy products contributed 10% TE (Figure 2).

In comparison with the recommendations [33], men had a ~70% higher intake of meat and ~60% higher intake of added oils/fats, ~40% lower intake of vegetables, ~80% lower intake of fruit, and very small intake of eggs, nuts, and seeds, while the intake of fish was almost absent. The intake of grains, dairy, sweets, and beverages/alcohol was adequate. Women had a ~70% higher intake of added oils/fats, ~30% higher intake of meat, ~20% higher intake of sweets, ~30% and ~50% lower intake of vegetables and fruit, respectively, and a very small intake of eggs and nuts/seeds, while the intake of fish was again almost absent. The intake of grains, dairy, and beverages/alcohol was adequate among women.

**Figure 3:**
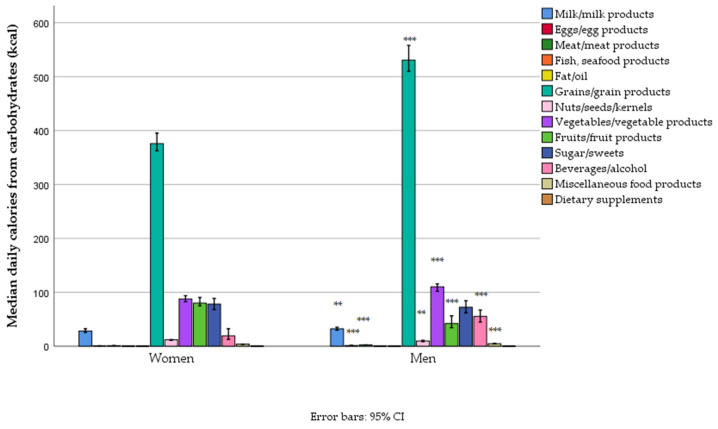
Carbohydrate-related energy intake from different food groups in women and men in a nationally representative sample of 18–64-year-old adults (*n* = 1139) living in Serbia (** *p* < 0.01, and *** *p* < 0.001).

Most of the carbohydrates came from grain products, with lesser amounts from vegetables, sugars/sweets, fruits, and beverages/alcohol. Overall, except for the intake of sugars/sweets, which was not different, and fruit, which was higher in women, men had a significantly higher carbohydrate intake across all other food groups (Supplementary Table S2, Figure 3).

**Figure 4:**
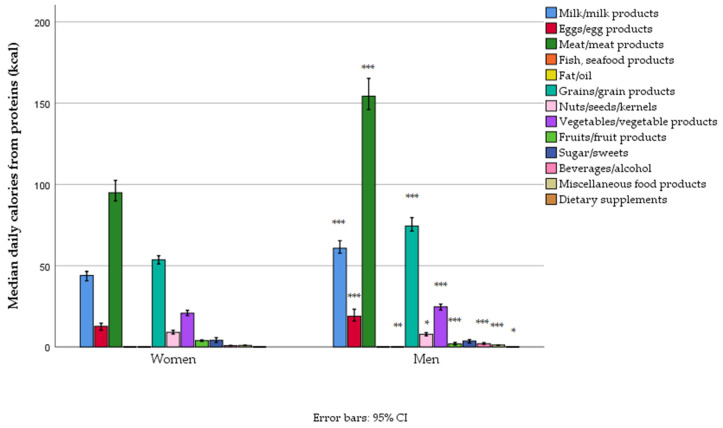
Protein-related energy intake from different food groups in women and men in a nationally representative sample of 18–64-year-old adults (*n* = 1139) living in Serbia (* *p* < 0.05, ** *p* < 0.01, and *** *p* < 0.001).

Most of the protein came from meat, then grain products and dairy, with lesser amounts from vegetables and eggs. Except for sweets and fish/seafood, protein intake from all other foods was significantly higher in men (Figure 4, Supplementary Table S3).

**Figure 5:**
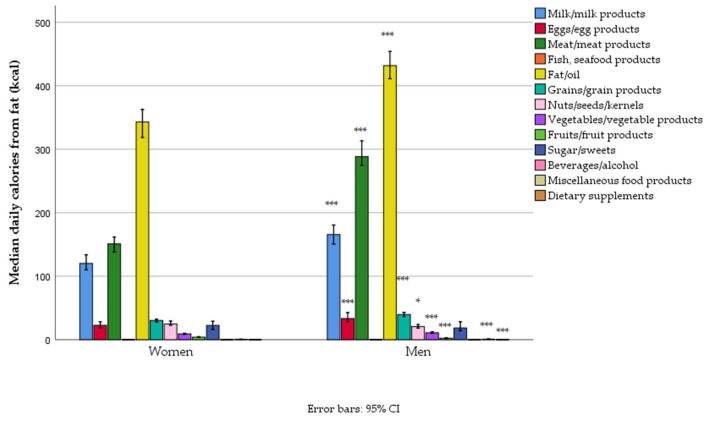
Fat-related energy intake from different food groups in women and men in a nationally representative sample of 18–64-year-old adults (*n* = 1139) living in Serbia (* *p* < 0.05 and *** *p* < 0.001).

Most of the fat came from added fats and oils, but also from meat and dairy products (Figure 5). Overall, except for fats/oils, meat and dairy products, women had higher fat intakes from nuts, seeds and kernels while men had more of their fat from grains and eggs (Supplementary Table S4).

## 4. Discussion

The global food system is encountering a multitude of challenges. These include climate change, resource scarcity, biodiversity loss, soil degradation, an increasing and ageing population, urbanization, food waste, and issues of food poverty, malnutrition, and overnutrition. Each of these factors directly affects human and planetary health and significantly contributes to non-communicable diseases (NCDs), global environmental changes, and social health inequalities [34].

The dietary habits and nutritional status of Serbian adults, as documented by this comprehensive survey, indicate critical imbalances and gender disparities that are consistent with broader trends observed in similar populations. The reliance on energy-dense, nutrient-poor foods, such as grains, fats, and meat, contrasts sharply with the insufficient consumption of fruits, vegetables, and dairy, deviating significantly from international dietary guidelines, such as the Dietary Guidelines for Americans (2020–2025) [31] or the recent WHO/FAO guidelines for healthy and sustainable diets from 2024 [29]. Furthermore, when compared to the recommendations for healthy and sustainable diets developed by the EAT-Lancet Commission on Healthy Diets from Sustainable Food Systems, the intake of health-promoting foods is too low while the intake of foods with high health and environmental impacts remains too high [32]. Globally, no region is on course to meet the Sustainable Development Goals (SDGs) aimed at limiting health and environmental burdens related to diets and the food system [35].

As a result, we observed increased percentages of overweight and obesity, 35% and 12%, respectively, where the WHO reported in 2022 57% and 23%, respectively [36]. The trend of overweigh/obesity rates increasing over the years has been observed in the majority of countries worldwide, including Serbia [37]. Similar trends are observed in some neighboring and other European countries: North Macedonia recorded 37% overweight and 19.6% obese (higher in men than in women) [38], in Italy it is slightly less—28.2% overweight and 12.5% obese (higher in men than in women)—while in Slovenia almost 60% of adults are overweight and obese [39], and in Poland 42% are overweight and 16.4% obese (higher in men than in women) [40]. In Serbia, a higher percentage of overweight and obese men correlate with observed dietary and lifestyle indicators: men have a higher percentage of low and medium physical activity levels compared to women, and 42% of men exceed the 3000 kcal/day energy intake, consume more alcohol, and their diet mostly consists of fat and protein sources and less carbohydrates, i.e., they consume more meat and less vegetables and fruits. All of this, combined with other lifestyle factors (screen time, sedentary jobs, lack of physical activity, smoking, etc.) contribute to the current nutritional status of men in Serbia. Further studies will look into the socioeconomic determinants of the nutritional status of the Serbian population.

One of the most concerning findings in this analysis is the generally low intake of fruits and vegetables, which contributed to only 397.6 g/day combined, despite their established role in providing essential micronutrients, fiber, and antioxidants [41]. This shortfall is perturbing given the WHO recommendation of consuming at least 400 g/day of fruits and vegetables to prevent non-communicable diseases (NCDs) [42]. Women consumed slightly more fruits and vegetables than men, but neither gender met the recommended levels, suggesting the need for targeted interventions to promote plant-based food consumption. Similar findings were found in Italy (313 g/day, combined) [43] and Slovenia (325.2 g/day, combined) [39], while a recent study in Bosnia and Herzegovina observed that the consumption of vegetables and fruits decreased with the increase in socioeconomic status [44]. Another study noted low fruit and vegetable consumption, below 400 g/day, in Denmark, the Czech Republic, Italy, and France. The average intake varied significantly: the Czech Republic population had lower intakes (118 g of fruit and 95 g of vegetables/day) while Italy had higher intakes (199 g of fruit and 239 g of vegetables/day). Women consistently had higher intakes than men. Elderly and highly educated individuals showed greater fruit intake in all four countries, but there was no difference according to overweight status. Vegetable intake was higher among the elderly in Denmark and France, among highly educated individuals in Denmark and the Czech Republic, and among overweight individuals in Italy and France [45].

The over-reliance on grains, particularly refined grains, as the primary carbohydrate source, and the underutilization of fiber-rich alternatives, like fruits, vegetables, and whole grains, also warrants attention. These dietary patterns contribute to an inadequate dietary fiber intake, with 68% of the population consuming less than 25 g/day, the minimum recommended for optimal health [46]. Likewise, two thirds of European countries do not even meet the lower recommended intake of 50 g [11]. For instance, in a Slovenian dietary Survey supported by EU Menu projects, it was observed that 89.6% adults had an inadequate dietary fiber intake, and that the average intake was 21 g/day [47]. In North Macedonia, the intake of dietary fibers was in the range of 14–29 g/day, depending on age and gender [38], while in Denmark, the Czech Republic, France, and Italy it was in range of 15.8–19.4 g/day [45]. There are actual Wholegrain Diet Initiatives that can be adapted to local settings by raising awareness and increasing the accessibility and affordability of wholegrain quality food products as well as switching to whole grains [48].

Protein intake was adequate for most participants, with men deriving significantly more energy from protein-rich foods, like meat, than women. Nevertheless, meat is also a rich source of fat, particularly saturated fat, which can lead to an energy surplus and metabolic consequences. Additionally, it was shown that red meat consumption is associated not only with the risk of cardiovascular diseases (CVDs) and diabetes [49] but also certain cancer forms, as well as mortality from those diseases [33,50]. Particularly, processed meat is associated with increased cancer risks (colorectal carcinoma) due to the formation of nitrosamines [51]. Studies show that a 30% reduction in the intake of red meat [49] significantly lowers the morbidity and mortality risk of carcinoma, CVDs, or diabetes and the socioeconomic burden from these diseases, indicating that sustainable diets not only benefit animals and the environment, but also all of society and consumers.

Serbia is one of the top meat-consuming countries in the world, together with some other countries in Europe [52,53]. Particularly, Central and Eastern Europe consume 121 g of unprocessed and 57 g of processed meat daily, which is double and triple, respectively, the amount of the world average [53].

However, despite the average adequate protein intake, approximately ~17%% of the population failed to meet the recommended protein intake of 0.83 g/kg body weight/day, with women disproportionately affected. This highlights a need for strategies to increase protein intake, particularly from diverse sources, such as legumes, nuts, and seeds, to enhance dietary variety, mitigate gender disparities, and also offer sustainable, plant-based dietary options [54,55].

Fat intake was excessive across the whole population: ~90%TE of the total population, surpassing the EFSA and US recommended levels of 20–35%TE, but the number of those who exceed the limits of the stricter recent WHO/FAO recommendations (15–30%TE) was much higher, 97% in both women and men [29,30]. Men consumed more fat from animal sources, such as meat and dairy, although added oils and fats (in first place was an omega-6-rich source—industrially refined sunflower oil) were the highest contributors.

A similar pattern was observed among women, but with slightly less animal fats. Comparable findings were observed in other European surveys, where almost all men exceeded the WHO upper limit for fat (30%) [3]. In our study, women similarly exceeded 30%, which can indicate that the goal of the WHO/FAO guidelines is not achievable in our population. Indeed, even in their recommendations [30], the WHO/FAO experts limit their recommendations as “conditional” and state that: “higher intakes are acceptable provided energy balance is maintained and saturated fat limits are not exceeded”. However, in our population, we can expect that saturated fat intakes are above the limits and the energy balance is not achieved, since there is a high prevalence of overweight, particularly among the male population.

There are health indices that suggest a potential relationship between an inadequate intake of various types of fatty acids and their effects on health [56]. Unfortunately, the data on actual fatty acid intakes in the general adult population are currently lacking [57], but our search guides us toward the conclusion that not only the amount but also the quality of fat intake is highly compromised. Nevertheless, further studies are needed to examine the quality (i.e., composition) of fat consumed in Serbia, by examining the dietary intakes of particular fatty acids and comparing them with the biomarkers of the intake at the population level.

Diets rich in omega-6 sources are recognized as pro-inflammatory and detrimental to health, connected to higher morbidity and mortality related to CVDs, carcinoma, and COVID-19, not only because of the increased risk of an energy surplus, but also because of the imbalance between Ω-3 and Ω-6 fatty acids [58,59]. In our population, the consumption of physiologically active, very-long-chain Ω-3 fatty acids (which derive from marine sources, less from fortified eggs and other animal sources) is very low compared to the consumption of their precursor from plant sources (flax-seed oil, flaxseeds, walnuts, and chia). According to the recent study, Serbia is one of the countries with the lowest fish consumption levels in Europe [53]. Additionally, the conversion of Ω -3 precursors in the body is very low and can be reduced even more by an unhealthy lifestyle and some health conditions (which are common in our population, e.g., obesity, diabetes, smoking, physical inactivity, and high Ω-6 diets) [60,61]. Therefore, bearing in mind such a high intake of omega-6 sources (through sunflower oil), which was much higher than the consumption of plant and animal sources of omega-3, we can expect the ratio of Ω-3 to Ω-6 to be much below the required level. Indeed, several recent studies in the Serbian general adult population show that the ratio of omega-3 to omega-6 in human blood is much lower than the required level [60]. The negative effect of certain saturated fatty acids is well known, while the intake of highly saturated fat sources increased in our population (not only through animal sources, but also through hydrogenated fats) [62,63]. The intake of sources of trans-fatty acids was also high in our population, through various industrial products with added margarines, and the research confirms the high levels of trans-fatty acids in Serbian staple foods [64]. Milk fat is also an important source, and some researchers highlight the negative health effects of high-fat milk products, bearing in mind the abundance of saturated and trans-fatty acids [65]. Nevertheless, some new studies question their foundation [66] and more research is needed.

A survey conducted at the beginning of the COVID-19 crisis, specifically during lockdown in several European countries, including Serbia, observed a significant increase in the consumption of Mediterranean diet ingredients (olive oil, nuts, fish, legumes, fruits, vegetables, white meat, etc.). These foods contain important nutrients crucial for maintaining the immune system, such as olive oil and omega-3 fatty acids (from fish), vitamin D, and antioxidants like vitamins C, E, and beta-carotene from fruits and vegetables. Additionally, there was an increase in home-cooking with these foods perceived as healthy, such as fresh vegetables [67].

Overall, men’s diet is characterized by a higher total energy intake and greater reliance on meat and fats, contributing to an elevated risk of obesity and related NCDs [68]. Conversely, women tended to consume less energy overall compared to men, which contributed to a higher likelihood of not meeting the recommended intake levels for essential nutrients, like protein and fiber, which could increase their risk of inadequate nutrition. These gender-specific differences in energy and macronutrient intake emphasize the need for tailored dietary guidelines, targeted educational campaigns, and other interventions to address unique dietary needs and challenges. Moreover, previous studies demonstrated a direct correlation between a low socioeconomic status and demographic indicators and the prevalence of all NCDs. The most vulnerable population groups are women, the elderly, and low-economic-status individuals, and they are the most prone to the development of NCDs [8], which is in line with some indicators presented in this study as well. It has been estimated that Serbia has ≥397 deaths per 100,000 population and 6684 to <8740 DALY rate per 100,000 population attributable to diet (high in sodium and low in fruits and wholegrains as major factors) in 2017, which is among the highest in the world.

### 4.1. Recommendations for Public Health Strategies to Target Gender-Specific Nutritional Challenges

To improve dietary patterns in Serbia, a multi-pronged public health strategy is essential. Here are some tailor-made recommendations to address identified nutritional challenges in the population: 

1. Subsidization of Healthier Foods: Encouraging a shift toward consuming healthier, plant-based, and alternative protein sources requires public awareness campaigns and research-driven initiatives to understand and promote sustainable dietary behaviors. Integrating strategies for affordable, attractive, and safe food options can drive healthy and sustainable diets [48]. Such efforts must prioritize equitable access to diverse, high-quality foods that align with cultural preferences, simultaneously addressing malnutrition in all its forms while promoting environmental sustainability and long-term health [69]. This can be achieved through the introduction of subsidies for fresh fruits, vegetables, and whole grains, with a focus on making these foods more affordable in low-income and rural areas and targeted financial incentives for producers of healthy foods, such as locally grown, organic produce, to support local economies while improving dietary intake. We observed a steady increase in plant-based-diet practitioners and studied their nutritional status through the EFSA EU Menu Survey [70], as well as accessibility and offers of vegan/vegetarian/plant-based/clean label food options in health food stores, groceries, restaurants, and catering services in Serbia in the past several years. 

2. Gender-Specific Nutrition Education and Awareness Campaigns are essential for addressing the dietary needs and preferences unique to each gender. These differences often arise from cultural, social, and economic factors, with women generally having a greater awareness of nutritional guidelines, while men may show a greater preference for energy-dense, nutrient-poor foods. To bridge this gap, it is crucial to tailor public health campaigns to focus on these distinct gender-based dietary patterns and obstacles, ensuring that each group receives the support and education they need. A key policy recommendation is to launch media campaigns that emphasize the importance of balanced diets, with a specific focus on, for instance, women and the importance of iron and calcium, while men may need a greater emphasis on protein and fiber intake. Moreover, it is vital to develop educational programs in workplaces that promote the benefits of a balanced diet.

3. Introduce workplace wellness initiatives that promote balanced meal options in cafeterias and vending machines, ensuring that both men and women have access to healthier food choices. Organize gender-tailored nutrition workshops and wellness challenges (e.g., a “Healthy Eating Challenge” with a focus on reducing sugar and increasing fiber intake). Provide incentives for employees to participate in wellness programs that include nutrition counseling or weight management support, with a particular emphasis on gender-specific health risks (e.g., women’s risk of osteoporosis and men’s higher rates of heart disease). 

4. Food Labeling and Public Awareness: Introduce mandatory front-of-pack labeling that highlight key nutrients (e.g., calories, fat, sugar, and fiber) to enable consumers to make informed choices at the point of purchase. Labels should include information tailored to gender-specific nutritional needs.

### 4.2. Limits and Strengths

The limitation of dietary intake surveys and subsequent data analysis is that these surveys typically categorize foods into broad groups, which may not fully capture the diversity and nuances of individual diets or account for variations in food preparation and consumption processes. Furthermore, the reliance on self-reported data can introduce recall bias or inaccuracies to portion size estimations. A cross-sectional design provides a snapshot of dietary habits but cannot assess trends or causality while limiting granularity in food group subcategories, e.g., differences between refined and whole grains; the analysis focuses more on the current trends rather than long-term changes or causal relationships. Thus, efforts were made to minimize these limitations through rigorous training for interviewers, careful food group classification, and validation of data collection methods and tools, such as a validated Food Atlas for Balkan countries [22], to ensure the accuracy and reliability of the findings. Despite these limitations, this study provides critical and epochal insights into the dietary patterns of Serbian adults and highlights priority areas for future research.

The previous national dietary survey, conducted more than twenty years ago, was the basis for the development of contemporary methodology, especially for the organization of food composition data collection and algorithms of dietary intake calculations. Yet, many improvements have been made. The YUSAD study used a 7-day food consumption record for the whole household, while this study adopted 2X 24HDR for an individual examinee, as a golden standard for the dietary intake assessment [71]. Food composition data are collected in a harmonized way, following the EuroFIR^TM^ standards [23]. Internationally validated and proved dietary intake assessment tools were used [17].

This study’s strengths are in its comprehensive, nationally representative sample, adherence to the EFSA EU Menu guidelines, and detailed data on dietary intake and anthropometric measures. Seasonal and weekday distributions of data collection enhanced reliability, and gender-specific analyses provided valuable insights for targeted interventions. Rigorous quality assurance ensured data integrity, and this study’s alignment with the international standards enabled cross-country comparisons [72]. This study enhances the research capabilities across the Balkan region allowing the monitoring of the effects of food policy actions in the field of governance and public health nutrition, the food supply chain and food environment, consumer behavior in the Belgrade declaration [73] on the development of sustainable food systems for healthy diets, and nutrition in Central and Southeastern Europe [74].

Since Serbia is a country without national dietary guidelines and nutrient recommendations, these results present the best evidence and basis for further developments of contemporary FAO Food System-based Dietary Guidelines with the holistic nutrition-based, sustainable, and field-to-fork approach in which the specific dietary challenges of the nation shall be addressed following the knowledge of the benefits of Mediterranean and Nordic diet models [73,74].

Finally, this survey, along with its data, methodological resources, and the comprehensive research infrastructure, is essential for establishing effective science–policy interfaces (SPIs) that bridge local and global food systems in a coordinated manner [46,75]. These efforts are aligned with the EU Strategic Research and Innovation Agenda (SRIA), which emphasizes the need for integrated research, innovation, and governance mechanisms to address food system challenges. Furthermore, these accomplishments serve as valuable assets for advancing the research and innovation within the framework of the Partnership for Sustainable Food Systems for People, Planet, and Climate, which aims to mobilize a transdisciplinary collaboration and perform various actions across the food system to accelerate its transformation [76].

## 5. Conclusions

This study provides an overview of dietary intake and nutritional status among Serbian adults. It highlights the excessive consumption of energy-dense, nutrient-poor foods, and the insufficient intake of fiber, fruits, vegetables, and dairy, leading to a higher risk of non-communicable diseases. Notably, men consume more fats and total energy, while women often lack an adequate protein and fiber intake.

The findings underscore the need for tailored nutritional policies and guidelines to improve public health and offer insights into Serbian dietary behavior data for developing evidence-based dietary guidelines for Serbia, shaping policies to foster healthier food environments. Its findings contribute to the national health strategies and global efforts for sustainable, equitable food systems, aiming to improve public health and reduce diet-related diseases.

Addressing these dietary challenges requires a comprehensive approach, including policy incentives for consuming fruits, vegetables, and whole grains; regulatory actions to reduce fat, salt, and sugar intake; and public awareness campaigns to promote sustainable dietary habits, like consuming plant-based proteins. Technological innovations, such as digital dietary tracking and AI-driven nutritional assessments, can enhance dietary monitoring and interventions.

## Figures and Tables

**Table 1:** Socio-demographic characteristics of the study population.

Variable	Total Sample *n* = 1139	Women *n* = 576	Men *n* = 563	*p*
**Response rate, %**	80.7	81.8	79.5	
**Age, years, median (IQR)**	42 (29–52)	43.0 (29–52)	41 (29–51)	0.351
**Age group, *n* (%)**				0.549
18–24 years	149 (13.1%)	72 (12.5%)	77 (13.7%)	
25–44 years	468 (41.1%)	231 (40.1%)	237 (42.1%)	
45–64 years	522 (45.8%)	273 (47.4%)	249 (44.2%)	
**Distribution per geographical region, *n* (%)**				0.912
Belgrade (capital city) region	270 (23.7%)	141 (24.5%)	129 (22.9%)	
Vojvodina region	307 (27.0%)	156 (27.1%)	151 (26.8%)	
Region of Šumadija and Western Serbia	320 (28.1%)	160 (27.8%)	160 (28.4%)	
Southeastern Serbia region	242 (21.2%)	119 (20.7%)	123 (21.8%)	
**Settlement type, *n* (%)**				0.527
Urban	975 (85.6%)	496 (86.1%)	479 (85.1%)	
Rural	164 (14.4%)	80 (13.9%)	84 (14.9%)	
**Ethnicity, *n* (%)**				0.991
Serbian	1060 (93.1%)	536 (93.1%)	524 (93.1%)	
Other	79 (6.9%)	40 (6.9%)	39 (6.9%)	
**Religion, *n* (%)**				0.803
Orthodox	1025 (90.0%)	517 (89.8%)	508 (90.2%)	
Catholic	36 (3.2%)	21 (3.6%)	15 (2.7%)	
Islamic	14 (1.2%)	7 (1.2%)	7 (1.2%)	
Other	64 (5.6%)	31 (5.4%)	33 (5.9%)	
**Marital status, *n* (%)**				0.004 **
Single	378 (33.2%)	173 (30.0%)	205 (36.4%)	
Married	602 (52.9%)	304 (52.8%)	298 (52.9%)	
Divorced	57 (5.0%)	36 (6.2%)	21 (3.7%)	
Separated	21 (1.8%)	11 (1.9%)	10 (1.8%)	
Single parent	1 (0.1%)	0 (0.0%)	1 (0.2%)	
Widowed	36 (3.2%)	28 (4.9%)	8 (1.4%)	
Other	44 (3.9%)	24 (4.2%)	20 (3.6%)	
**Household size and composition, median (IQR)**				
People per household	30 (2–4)	3 (2–4)	3 (2–4)	0.826
**Highest level of formal education, *n* (%)**				0.007 **
ISCED 0: less than primary educational attainment	7 (0.6%)	4 (0.7%)	3 (0.5%)	
ISCED 1: Primary education	30 (2.6%)	17 (3.0%)	13 (2.3%)	
ISCED 2: Lower secondary education	46 (4.0%)	15 (2.6%)	31 (5.5%)	
ISCED 3: Upper secondary education	476 (41.8%)	233 (40.5%)	243 (43.2%)	
ISCED 4/5: Post-secondary/Short-cycle tertiary education	73 (6.4%)	29 (5.0%)	44 (7.8%)	
ISCED 6: Bachelor’s or equivalent level	359 (31.5%)	187 (32.5%)	172 (30.6%)	
ISCED 7/8: Master’s/Doctoral or equivalent level	148 (13.0%)	91 (15.8%)	57 (10.1%)	
**Employment status, *n* (%)**				<0.001 ***
Unemployed	81 (7.1%)	41 (7.1%)	40 (7.1%)	
Working for pay or profit	812 (71.3%)	382 (66.3%)	430 (76.4%)	
Pupil, student, further training, unpaid work experience	133 (11.7%)	77 (13.4%)	56 (9.9%)	
In retirement or early retirement or has given up business	59 (5.2%)	40 (6.9%)	19 (3.4%)	
Maternity, parental, or sick leave	9 (0.8%)	8 (1.4%)	1 (0.2%)	
Permanently disabled	4 (0.4%)	2 (0.3%)	2 (0.4%)	
Fulfilling domestic tasks	16 (1.4%)	15 (2.6%)	1 (0.2%)	
Not applicable/Other	25 (2.2%)	11 (1.9%)	14 (2.5%)	
**Professional profile, *n* (%)**				
Managers	92 (8.1%)	38 (6.6%)	54 (9.6%)	<0.001 ***
Professionals	253 (22.2%)	132 (22.9%)	121 (21.5%)	
Technicians and associate professionals	134 (11.8%)	58 (10.1%)	76 (13.5%)	
Clerical support workers	114 (10.0%)	91 (15.8%)	23 (4.1%)	
Service and sales workers	108 (9.5%)	52 (9.0%)	56 (9.9%)	
Skilled agricultural, forestry, and fishery workers	7 (0.6%)	3 (0.5%)	4 (0.7%)	
Craft and related trades workers	55 (4.8%)	19 (3.3%)	36 (6.4%)	
Plant and machine operators, and assemblers	35 (3.1%)	3 (0.5%)	32 (5.7%)	
Elementary occupations	10 (0.9%)	1 (0.2%)	9 (1.6%)	
Armed forces occupations	41 (3.6%)	12 (2.1%)	29 (5.2%)	
Other	289 (25.4%)	167 (29.0%)	122 (21.5%)	
**Suffer from chronic illness, *n* (%)**				
No	859 (75.4%)	400 (69.4%)	459 (81.5%)	<0.001 ***
Yes:	280 (24.6%)	176 (30.6%)	104 (18.5%)	
Neoplasms	6 (0.5%)	5 (0.9%)	1 (0.2%)	
Diseases of the blood-forming organs and immune system	11 (1.0%)	8 (1.4%)	3 (0.5%)	0.218
Endocrine, nutritional, and metabolic diseases	92 (8.1%)	74 (12.8%)	18 (3.2%)	0.225
Mental and behavioral disorders	11 (1.0%)	4 (0.7%)	7 (1.2%)	<0.001 ***
Diseases of the nervous system	8 (0.7%)	7 (1.2%)	1 (0.2%)	0.380
Diseases of the circulatory system	150 (13.2%)	85 (14.8%)	65 (11.5%)	0.069
Diseases of the respiratory system	35 (3.1%)	22 (3.8%)	13 (2.3%)	0.109
Diseases of the digestive system	22 (1.9%)	11 (1.9%)	11 (2.0%)	0.140
Diseases of the skin and subcutaneous tissue	4 (0.4%)	3 (0.5%)	1 (0.2%)	0.957
Diseases of the musculoskeletal system and connective tissue	19 (1.7%)	16 (2.8%)	3 (0.5%)	0.624
Diseases of the genitourinary system	13 (1.1%)	11 (1.9%)	2 (0.4%)	0.004 **
Other	3 (0.3%)	2 (0.3%)	1 (0.2%)	0.022 *
**Taking chronic medications, *n* (%)**				1.000
No	897 (78.8%)	420 (72.9%)	477 (84.7%)	<0.001 ***
Yes	242 (21.2%)	156 (27.1%)	86 (15.3%)	
**Smoking status, *n* (%)**				
Current smoker	358 (31.4%)	176 (30.6%)	182 (32.3%)	0.385
Former smoker	178 (15.6%)	84 (14.6%)	94 (16.7%)	
Never smoker	603 (52.9%)	316 (54.9%)	287 (51.0%)	
**Physical activity level, *n* (%)**				
Low	444 (39.0%)	198 (34.4%)	246 (43.7%)	<0.001 ***
Medium	567 (49.8%)	321 (55.7%)	246 (43.7%)	
High	128 (11.2%)	57 (9.9%)	71 (12.6%)	
**Physical activity METS, min/week, median (IQR)**	3756 (1746–7284)	3383 (1668–6703)	4185 (1851–7866)	
**Dietary pattern, *n* (%)**				0.006 **
Conventional	1074 (94.3)	528 (91.7)	546 (96.9)	0.004 **
Diet linked to health conditions (e.g., celiac disease, allergy, diabetes, obesity)	65.0 (5.7)	48 (8.3)	17 (3.0)	
**Season, *n* (%)**				
Fall	290 (25.5%)	149 (25.9%)	141 (25.0%)	0.983
Spring	285 (25.0%)	143 (24.8%)	142 (25.2%)	
Summer	283 (24.8%)	144 (25.0%)	139 (24.7%)	
Winter	281 (24.7%)	140 (24.3%)	141 (25.0%)	

METS—Metabolic equivalents of the task; IQR—interquartile range; *p*—statistical significance of difference (bolded values are statistically significant, * *p* < 0.05, ** *p* < 0.01, and *** *p* < 0.001); differences between men and women were tested with the Mann–Whitney test (for numerical data) and with chi-squared test or Fisher’s exact test (for categorical data).

**Table 2:** Anthropometric data and nutritional status based on BMI categories for a nationally representative sample of adults aged 18–64 years living in Serbia (*n* = 1139).

Anthropometric Indicators	Total	Women	Men	*p*
	Median (IQR)	Median (IQR)	Median (IQR)	
Body height (cm)	174 (167–180)	168 (164–172)	180 (176–186)	<0.001 *
Body mass (kg)	75 (64–87)	65 (59–74)	85 (77–94)	<0.001 *
BMI (kg/m^2^)	24.8 (22.2–27.8)	23.1 (21.0–26.1)	25.9 (23.7–28.6)	<0.001 *
Nutritional status	*n* (%)	*n* (%)	*n* (%)	
Underweight (<18 kg/m^2^)	29 (2.5%)	21 (3.6%)	8 (1.4%)	<0.001 *
Normal weight (18–24 kg/m^2^)	574 (50.4%)	366 (63.5%)	208 (36.9%)	
Overweight (25–30 kg/m^2^)	401 (35.2%)	136 (23.6%)	265 (47.1%)	
Obese (>30 kg/m^2^)	135 (11.9%)	53 (9.2%)	82 (14.6%)	

BMI—body mass index. IQR—interquartile range; *p*—statistical significance of difference (bolded values are statistically significant, * *p* < 0.001); differences between men and women were tested with the Mann–Whitney test (for numerical data) and with the chi-squared test or Fisher’s exact test (for categorical data).

**Table 3:** Daily energy and macronutrient intake across gender categories in a nationally representative sample of adults 18–64 years old living in Serbia (*n* = 1139).

Proximate Daily Intake	Total	Women	Men	*p*
	Median (IQR)	Median (IQR)	Median (IQR)	
**Total energy (kcal)**	2373.0 (1896.0–3013.0)	2069.5 (1683.8–2439.0)	2800.0 (2293.0–3367.0)	<0.001 ***
**Carbohydrates (g)**	218.2 (168.2–274.3)	194.8 (150.5–241.8)	247.2 (191.9–309.5)	<0.001 ***
**Carbohydrates (kcal)**	872.8 (673.0–1097.2)	779.1 (601.8–967.0)	988.7 (767.6–1238.0)	<0.001 ***
**Carbohydrates (%TE)**	37.1 (31.9–41.9)	38.2 (33.0–43.3)	36.0 (30.6–40.7)	<0.001 ***
**Protein (g)**	87.7 (69.7–110.8)	74.1 (61.4–90.1)	104.6 (83.2–129.5)	<0.001 ***
**Protein (kcal)**	350.9 (278.9–443.2)	296.2 (245.6–360.5)	418.6 (332.7–518.1)	<0.001 ***
**Protein (%TE)**	14.6 (12.8–16.8)	14.3 (12.5–16.6)	14.9 (13.0–17.0)	0.001 **
**Protein (g/kg body mass)**	1.2 (0.9–1.5)	1.1 (0.9–1.4)	1.2 (1.0–1.6)	<0.001 ***
**Protein intake categories ^1^, *n* (%)**				0.003 **
<0.83 g/kg body mass	196 (17.2%)	118 (20.5%)	78 (13.9%)	
≥0.83 g/kg body mass	943 (82.8%)	458 (79.5%)	485 (86.1%)	
**Fat (g)**	117.7 (88.5–152.2)	99.2 (78.2–126.1)	139.3 (109.4–167.0)	<0.001 ***
**Fat (kcal)**	1059.6 (796.5–1369.3)	892.7 (704.0–1135.0)	1254.1 (984.3–1520.7)	<0.001 ***
**Fat (%TE)**	44.7 (40.0–49.3)	44.4 (39.2–48.8)	45.3 (40.8–49.7)	0.011 *
**Alcohol (g)**	0.01 (0.0–8.2)	0.01 (0.00–0.07)	0.03 (0.00–13.68)	<0.001 ***
**Alcohol (kcal)**	0.00 (0.0–2.1)	0.00 (0.00–0.03)	0.01 (0.00–3.40)	<0.001 ***
**Alcohol (%TE)**	0.07 (0.0–59.0)	0.04 (0.00–0.52)	0.24 (0.00–98.50)	<0.001 ***
**Fiber (g)**	20.7 (15.7–27.3)	20.0 (14.9–26.2)	21.7 (16.5–28.4)	<0.001 ***
**Fiber intake categories ^1^, *n* (%)**				0.026 *
<25 g	772 (67.8%)	408 (70.8%)	364 (64.7%)	
≥25 g	367 (32.2%)	168 (29.2%)	199 (35.3%)	
**Fiber (kcal)**	41.4 (31.3–54.6)	40.0 (29.8–52.4)	43.4 (32.9–56.8)	<0.001 ***
**Fiber (%TE)**	0.02 (0.01–0.02)	0.02 (0.02–0.02)	0.02 (0.01–0.02)	<0.001 ***

^1^ EFSA (European Food Safety Authority). Dietary Reference Values for Nutrients—Summary Report. EFSA supporting publications. December 2017. Available at https://efsa.onlinelibrary.wiley.com. TE—total energy; IQR—interquartile range; *p*—statistical significance of difference (bolded values are statistically significant, * *p* < 0.05, ** *p* < 0.01, and *** *p* < 0.001); differences between men and women were tested with the Mann–Whitney test (for numerical data) and with the chi-squared test or Fisher’s exact test (for categorical data).

**Table 4:** Comparison of energy intake categories and the energy intake values from different macronutrients according to the EFSA ^1^ and US ^2^ dietary guidelines and the present study’s results.

	Total (*n* = 1139)	Women (*n* = 576)	Men (*n* = 563)	
	*n* (%)	*n* (%)	*n* (%)	*p*
**Total Energy Intake ^1,2^**				<0.001 *
<1600 kcal/day (women), <2000 kcal/day (men)	200 (17.6%)	114 (19.8%)	86 (15.3%)	
1600–2400 kcal/day (women), 2000–3000 kcal/day (men)	547 (48.0%)	305 (53.0%)	242 (43.0%)	
≥2400 kcal/day (women), ≥3000 kcal/day (men)	392 (34.4%)	157 (27.3%)	235 (41.7%)	
**Percentage of energy intake coming from carbohydrates ^1^**				<0.001 *
<45%	979 (86.0%)	473 (82.1%)	506 (89.9%)	
45–60%	157 (13.8%)	102 (17.7%)	55 (9.8%)	
>60%	3 (0.3%)	1 (0.2%)	2 (0.4%)	
**Percentage of energy intake coming from carbohydrates ^2^**				<0.001 *
<45%	979 (86.0%)	473 (82.1%)	506 (89.9%)	
45–65%	160 (14.0%)	103 (17.9%)	57 (10.1%)	
>65%	0 (0.0%)	0 (0.0%)	0 (0.0%)	
**Percentage of energy intake coming from proteins ^1,2^**				0.001 *
<10%	27 (2.4%)	23 (4.0%)	4 (0.7%)	
10–35%	1110 (97.5%)	552 (95.8%)	558 (99.1%)	
>35%	2 (0.2%)	1 (0.2%)	1 (0.2%)	
**Percentage of energy intake coming from fats ^1,2^**				0.073
<20%	3 (0.3%)	0 (0.0%)	3 (0.5%)	
20–35%	108 (9.5%)	62 (10.8%)	46 (8.2%)	
>35%	1028 (90.3%)	514 (89.2%)	514 (91.3%)	

^1^ EFSA (European Food Safety Authority). Dietary Reference Values for Nutrients—Summary Report. EFSA supporting publications. December 2017. Available at https://efsa.onlinelibrary.wiley.com/. ^2^ U.S. Department of Agriculture and U.S. Department of Health and Human Services. Dietary Guidelines for Americans, 2020–2025. 9th Edition. December 2020. Available at https://www.dietaryguidelines.gov/. *p*—statistical significance of difference (bolded values are statistically significant * *p* < 0.001); differences between men and women were tested with the chi-squared test or Fisher’s exact test.

**Table 5:** Consumption of food groups (in grams per day) across gender categories in a nationally representative sample of 18–64-year-old adults (*n* = 1139) living in Serbia.

Food Groups	Total	Women	Men	*p*
	Median (IQR)	Median (IQR)	Median (IQR)	
Milk/milk products (g)	207.5 (109.7–321.8)	186.8 (99.9–295.3)	229.5 (126.0–342.6)	<0.001 *
Eggs/egg products (g)	30.0 (7.4–70.2)	25.5 (6.7–62.8)	37.5 (8.4–85.2)	<0.001 *
Meat/meat products (g)	162.8 (97.8–251.5)	124.9 (73.1–183.5)	214.5 (140.4–317.0)	<0.001 *
Fish/seafood products (g)	0.0 (0.0–0.0)	0.0 (0.0–0.0)	0.0 (0.0–0.0)	0.098
Fat/oil (g)	43.9 (30.2–62.1)	39.5 (26.4–54.3)	49.0 (33.5–68.2)	<0.001 *
Grains/grain products (g)	199.0 (136.6–272.3)	167.2 (115.4–226.1)	238.8 (172.4–314.4)	<0.001 *
Nuts/seeds/kernels (g)	13.1 (4.8–25.0)	14.1 (5.2–26.5)	12.7 (4.1–23.4)	0.109
Vegetables/vegetable products (g)	273.6 (194.2–379.2)	259.2 (180.4–373.3)	286.3 (206.9–393.1)	<0.001 *
Fruits/fruit products (g)	124.0 (0.0–255.0)	155.5 (41.7–274.5)	96.5 (0.0–234.9)	<0.001 *
Sugar/sweets (g)	27.7 (5.5–57.7)	28.7 (6.7–58.3)	26.1 (4.3–57.7)	0.402
Beverages/alcohol (g)	1599.2 (1226.9–2087.8)	1488.9 (1142.4–1946.7)	1725.0 (1299.2–2200.6)	<0.001 *
Miscellaneous food products (g)	6.5 (4.2–9.7)	5.7 (3.8–9.0)	7.3 (5.0–10.7)	<0.001 *
Dietary supplements (g)	0.0 (0.0–0.0)	0.0 (0.0–0.0)	0.0 (0.0–0.0)	0.126

IQR—interquartile range; *p*—statistical significance of difference (bolded values are statistically significant, * *p* < 0.001); differences between men and women were tested with the Mann–Whitney test.

**Table 6:** Contribution of food groups to total energy intake across gender categories in a nationally representative sample of 18–64-year-old adults (*n* = 1139) living in Serbia.

Food Groups (%TE)	Total	Women	Men	*p*
	Median (IQR)	Median (IQR)	Median (IQR)	
Milk/milk products	10.0 (5.8–14.1)	9.7 (5.7–14.2)	10.2 (6.0–13.9)	0.739
Eggs/egg products	1.8 (0.5–4.8)	1.6 (0.5–4.7)	2.00 (0.5–4.8)	0.384
Meat/meat products	14.5 (9.2–21.2)	12.6 (7.5–18.7)	17.0 (11.4–23.8)	<0.001 ***
Fish/seafood products	0.00 (0.00–0.00)	0.00 (0.00–0.00)	0.00 (0.00–0.00)	0.049 *
Fat/oil	16.4 (11.9–21.0)	17.1 (12.1–21.9)	15.7 (11.8–20.6)	0.014 *
Grains/grain products	23.9 (18.6–30.5)	23.2 (17.7–30.9)	24.6 (19.3–30.2)	0.071
Nuts/seeds/kernels	2.2 (0.7–4.9)	2.8 (1.1–6.1)	1.6 (0.5–3.8)	<0.001 ***
Vegetables/vegetable products	6.2 (4.0–9.3)	6.6 (4.1–9.8)	5.9 (3.8–8.7)	0.002 **
Fruits/fruit products	3.3 (0.0–7.1)	4.6 (1.1–8.5)	1.9 (0.0–5.5)	<0.001 ***
Sugar/sweets	4.6 (1.0–10.2)	5.6 (1.4–11.8)	4.0 (0.6–8.5)	<0.001 ***
Beverages/alcohol	2.7 (0.2–6.8)	1.8 (0.1–5.2)	3.9 (0.5–8.3)	<0.001 ***
Miscellaneous food products	0.28 (0.16–0.43)	0.30 (0.16–0.45)	0.26 (0.15–0.41)	0.025 *
Dietary supplements	0.00 (0.00–0.00)	0.00 (0.00–0.00)	0.00 (0.00–0.00)	0.206

TE—total energy; IQR—interquartile range; *p*—statistical significance of difference (bolded values are statistically significant, * *p* < 0.05, ** *p* < 0.01, and *** *p* < 0.001); differences between men and women were tested with the Mann–Whitney test.

## Data Availability

The original contributions presented in this study are included in the article/Supplementary Materials. Further inquiries can be directed to the corresponding author.

## Supplementary Materials

The following supporting information can be downloaded at: https://www.mdpi.com/article/10.3390/foods14071228/s1, Supplementary Table S1: Total daily energy intake derived from different food groups across gender categories in a nationally representative sample of 18–64-year-old adults (*n* = 1139) living in Serbia; Supplementary Table S2: Carbohydrate-related energy intake derived from different food groups across gender categories in a nationally representative sample of 18–64-year-old adults (*n* = 1139) living in Serbia; Supplementary Table S3: Protein-related energy intake derived from different food groups across gender categories in a nationally representative sample of 18–64-year-old adults (*n* = 1139) living in Serbia; Supplementary Table S4: Fat-related energy intake derived from different food groups across gender categories in a nationally representative sample of 18–64-year-old adults (*n* = 1139) living in Serbia.

## Abbreviations

The following abbreviations are used in this manuscript:

**AP**	Autonomous Province
**BMI**	Body Mass Index
**CAPNUTRA**	Capacity Development in Nutrition
**COVID-19**	Coronavirus Disease 2019
**CVDs**	Cardiovascular Diseases
**DALYs**	Disability-Adjusted Life Years
**DAP**	Diet Assess and Plan
**DRIs**	Dietary Reference Intakes
**DRVs**	Dietary Reference Values
**EAT-Lancet**	EAT-Lancet Commission on Healthy Diets from Sustainable Food Systems
**EFSA**	European Food Safety Authority
**EFSA EU Menu**	EFSA-coordinated harmonized food consumption survey
**EU**	European Union
**EU Menu**	European Union Menu food consumption survey
**FAO**	Food and Agriculture Organization
**FCDB**	Food Composition Database
**GBD**	Global Burden of Disease
**GDA**	Guideline Daily Amount
**GDP**	Gross Domestic Product
**IPAQ-SF**	International Physical Activity Questionnaire—Short Form
**IQR**	Interquartile Range
**ISCED**	International Standard Classification of Education
**METs**	Metabolic Equivalents of the Task
**NCDs**	Non-Communicable Diseases
**OMICS**	Comprehensive study of biological molecules
**PRI**	Population Reference Intake
**RDA**	Recommended Dietary Allowances
**SDG**	Sustainable Development Goal
**SPSS**	Statistical Package for the Social Sciences
**TE**	Total Energy
**TEI**	Total Energy Intake
**UN**	United Nations
**WHO**	World Health Organization
