# Supplementary material for: Energy and Macronutrient Dietary Intakes of Serbian Adults 18–64 Years Old: EFSA EU Menu Food Consumption Survey in Serbia (2017–2022)
Source: Foods. 2025 Mar 31;14(7):1228. doi: 10.3390/foods14071228 (PMC11988697; doi:10.3390/foods14071228)
Supplement: Supplementary file 1 [file foods-14-01228-s001.zip › foods-3480494-supplementary.pdf]

Supplementary Materials

Supplementary Table S1. Total daily energy intake deriving from different food groups across gender categories in a nationally representative sample of 18-64 years old adults (n=1139) living in Serbia.

Food groups (kcal/day)	Total	Women	Men	*p*
	Median (IQR)	Median (IQR)	Median (IQR)	
**Milk/milk products**	**233.1 (132.4-368.4)**	**202.4 (110.5-306.6)**	**269.30 (153.7-412.4)**	**<0.001*****
**Eggs/egg products**	43.8 (11.1-123.0)	36.2 (9.5-98.6)	53.3 (12.0-135.2)	**<0.001*****
**Meat/meat products**	**335.5 (197.6-542.7)**	**254.6 (150.1-362.8)**	**465.8 (292.9-693.3)**	**<0.001*****
**Fish/seafood products**	0.00 (0.00-0.00)	0.00 (0.00-0.00)	0.00 (0.00-0.00)	0.100
**Fat/oil**	**387.1 (264.7-541.7)**	**342.9 (233.4-467.4)**	**431.9 (300.9-600.8)**	**<0.001*****
**Grains/grain products**	**573.3 (397.8-777.5)**	**484.1 (342.7-656.8)**	**684.6 (478.6-878.2)**	**<0.001*****
**Nuts/seeds/kernels**	50.5 (15.1-107.0)	57.6 (20.1-114.7)	47.6 (11.5-98.8)	**0.026***
**Vegetables/vegetable products**	149.3 (93.5-220.2)	135.0 (86.2-203.3)	167.7 (100.6-242.3)	**<0.001*****
**Fruits/fruit products**	82.6 (0.0-159.7)	96.6 (22.9-166.5)	50.0 (0.0-152.8)	**<0.001*****
**Sugar/sweets**	114.7 (21.5-237.5)	117.2 (25.4-235.3)	111.2 (17.4-241.8)	0.412
**Beverages/alcohol**	66.1 (3.9-172.0)	41.5 (2.5-109.7)	107.5 (10.3-249.2)	**<0.001*****
**Miscellaneous food products**	6.8 (3.8-10.4)	6.2 (3.4-9.2)	7.7 (4.2-11.6)	**<0.001*****
**Dietary supplements**	0.00 (0.00-0.00)	0.00 (0.00-0.00)	0.00 (0.00-0.00)	0.194

IQR – interquartile range; *p* – statistical significance of difference (bolded values are statistically significant, *$p<0.05$, **$p<0.01$, ***$p<0.001$); Differences between men and women were tested with the Mann-Whitney test.

Supplementary Table S2. Carbohydrate-related energy intake deriving from different food groups across gender categories in a nationally representative sample of 18-64 years old adults (n=1139) living in Serbia.

**Food groups (kcal carbohydrates/day)**	**Total**	**Women**	**Men**	*p*
	**Median (IQR)**	**Median (IQR)**	**Median (IQR)**	
**Milk/milk products**	30.8 (14.9-50.5)	28.3 (12.8-47.3)	32.3 (16.6-53.6)	**0.004****
**Eggs/egg products**	1.0 (0.3-2.2)	0.8 (0.2-2.0)	1.2 (0.3-2.7)	**<0.001*****
**Meat/meat products**	1.6 (0.1-4.0)	1.1 (0.0-2.5)	2.5 (0.7-5.1)	**<0.001*****
**Fish/seafood products**	0.00 (0.00-0.00)	0.00 (0.00-0.00)	0.00 (0.00-0.00)	0.130
**Fat/oil**	0.04 (0.00-0.20)	0.03 (0.00-0.20)	0.04 (0.00-0.21)	0.421
**Grains/grain products**	443.3 (310.2-604.5)	375.8 (258.9-506.1)	530.7 (381.3-681.0)	**<0.001*****
**Nuts/seeds/kernels**	11.0 (2.9-19.8)	12.0 (4.1-21.3)	9.6 (2.1-17.9)	**0.006****
**Vegetables/vegetable products**	97.9 (58.6-144.5)	87.6 (53.0-129.3)	110.3 (64.8-155.6)	**<0.001*****
**Fruits/fruit products**	68.4 (0.0-137.0)	80.0 (17.8-144.0)	41.9 (0.0-128.9)	**<0.001*****
**Sugar/sweets**	76.4 (19.0-155.7)	78.2 (22.0-156.8)	72.0 (14.2-151.1)	0.358
**Beverages/alcohol**	41.0 (1.8-102.4)	19.2 (1.0-72.9)	55.5 (3.1-137.6)	**<0.001*****
**Miscellaneous food products**	4.2 (2.4-6.4)	3.8 (2.1-5.5)	4.7 (2.7-7.0)	**<0.001*****
**Dietary supplements**	0.00 (0.00-0.00)	0.00 (0.00-0.00)	0.00 (0.00-0.00)	0.212

IQR – interquartile range; *p* – statistical significance of difference (bolded values are statistically significant, *$p<0.05$, **$p<0.01$, ***$p<0.001$); Differences between men and women were tested with the Mann-Whitney test.

Supplementary Table S3. Protein-related energy intake deriving from different food groups across gender categories in a nationally representative sample of 18-64 years old adults (n=1139) living in Serbia.

Food groups (kcal protein/day)	Total	Women	Men	*p*
	Median (IQR)	Median (IQR)	Median (IQR)	
**Milk/milk products**	51.3 (28.5-79.7)	44.0 (24.6-67.9)	60.8 (35.1-91.4)	**<0.001*** **
**Eggs/egg products**	14.9 (3.9-37.6)	12.8 (3.4-34.1)	18.9 (4.3-43.7)	**<0.001*** **
**Meat/meat products**	118.4 (70.4-186.3)	94.9 (53.2-140.1)	154.3 (97.8-240.6)	**<0.001*** **
**Fish/seafood products**	0.00 (0.00-0.00)	0.00 (0.00-0.00)	0.00 (0.00-0.00)	0.097
**Fat/oil**	0.04 (0.00-0.18)	0.03 (0.00-0.14)	0.05 (0.00-0.21)	**0.007** **
**Grains/grain products**	63.8 (44.4-85.3)	53.6 (38.0-72.7)	74.4 (54.9-96.6)	**<0.001*** **
**Nuts/seeds/kernels**	8.2 (2.7-16.0)	9.0 (3.5-17.7)	7.8 (1.7-14.4)	**0.013* **
**Vegetables/vegetable products**	22.6 (14.0-38.1)	20.8 (13.3-33.7)	24.7 (15.2-42.2)	**<0.001*** **
**Fruits/fruit products**	3.2 (0.0-6.6)	3.9 (0.8-7.3)	1.9 (0.0-5.8)	**<0.001*** **
**Sugar/sweets**	3.7 (0.0-13.8)	4.2 (0.0-13.9)	3.7 (0.0-13.8)	0.319
**Beverages/alcohol**	1.7 (0.1-5.0)	0.8 (0.0-3.1)	2.0 (0.2-7.6)	**<0.001*** **
**Miscellaneous food products**	1.1 (0.7-1.7)	0.9 (0.5-1.5)	1.2 (0.6-1.8)	**<0.001*** **
**Dietary supplements**	0.00 (0.00-0.00)	0.00 (0.00-0.00)	0.00 (0.00-0.00)	**0.020* **

IQR – interquartile range; *p* – statistical significance of difference (bolded values are statistically significant, **p*<0.05, ***p*<0.01, ****p*<0.001); Differences between men and women were tested with the Mann-Whitney test.

Supplementary Table S4. Fat-related energy intake deriving from different food groups across gender categories in a nationally representative sample of 18-64 years old adults (n=1139) living in Serbia.

Food groups (kcal fat/day)	Total	Women	Men	*p*
	Median (IQR)	Median (IQR)	Median (IQR)	
**Milk/milk products**	139.8 (72.4-237.7)	120.2 (61.1-197.7)	165.6 (92.1-278.9)	**<0.001*** **
**Eggs/egg products kcal**	28.2 (6.4-77.7)	22.7 (5.6-62.4)	33.4 (7.4-91.0)	**<0.001*** **
**Meat/meat products**	207.5 (113.6-352.9)	150.9 (79.1-229.9)	288.4 (171.2-461.2)	**<0.001*** **
**Fish/seafood products**	0.00 (0.00-0.00)	0.00 (0.00-0.00)	0.00 (0.00-0.00)	0.106
**Fat/oil**	385.7 (264.7-539.9)	343.0 (230.1-467.1)	431.8 (300.9-600.7)	**<0.001*** **
**Grains/grain products**	34.8 (20.1-61.2)	30.4 (17.3-53.8)	39.7 (23.6-69.5)	**<0.001*** **
**Nuts/seeds/kernels**	23.3 (6.4-66.4)	24.8 (8.3-70.5)	20.7 (5.8-62.4)	**0.040***
**Vegetables/vegetable products**	10.1 (5.6-19.4)	9.2 (5.21-16.95)	10.9 (6.2-20.5)	**<0.001*** **
**Fruits/fruit products**	3.1 (0.0-6.8)	4.1 (1.01-7.71)	2.4 (0.0-5.9)	**<0.001*** **
**Sugar/sweets**	21.1 (0.00-75.4)	22.6 (0.0-73.9)	18.6 (0.0-76.3)	0.469
**Beverages/alcohol**	0.02 (0.00-1.71)	0.02 (0.00-1.80)	0.13 (0.00-1.70)	0.770
**Miscellaneous food products**	0.81 (0.33-1.54)	0.72 (0.28-1.27)	0.96 (0.40-1.73)	**<0.001*** **
**Dietary supplements**	0.00 (0.00-0.00)	0.00 (0.00-0.00)	0.00 (0.00-0.00)	**0.021***

IQR – interquartile range; *p* – statistical significance of difference (bolded values are statistically significant, **p*<0.05, ***p*<0.01, ****p*<0.001); Differences between men and women were tested with the Mann-Whitney test.
